# The Role of Cone Beam Computed Tomography in Periodontology: From 3D Models of Periodontal Defects to 3D-Printed Scaffolds

**DOI:** 10.3390/jpm14020207

**Published:** 2024-02-14

**Authors:** Styliani Verykokou, Charalabos Ioannidis, Sofia Soile, Christos Angelopoulos, Konstantinos Theodoridis, Athanasios S. Arampatzis, Andreana N. Assimopoulou, Dimitrios Christofilos, Afroditi Kapourani, Ioannis Pantazos, Panagiotis Barmpalexis, Argyro-Maria Boutsi, Chryssy Potsiou

**Affiliations:** 1 Laboratory of Photogrammetry, School of Rural, Surveying and Geoinformatics Engineering, National Technical University of Athens, 15780 Athens, Greece; cioannid@survey.ntua.gr (C.I.); ssoile@survey.ntua.gr (S.S.); iboutsi@mail.ntua.gr (A.-M.B.); chryssyp@survey.ntua.gr (C.P.); 2 Department of Oral Diagnosis and Radiology, School of Dentistry, National and Kapodistrian University of Athens, 11527 Athens, Greece; cangelopou@dent.uoa.gr; 3 Laboratory of Organic Chemistry, School of Chemical Engineering, Aristotle University of Thessaloniki, 54124 Thessaloniki, Greece; tgkonsta@auth.gr (K.T.); arampatzisa@cheng.auth.gr (A.S.A.); adreana@gapps.auth.gr (A.N.A.); 4 School of Chemical Engineering & Physics Laboratory, Faculty of Engineering, Aristotle University of Thessaloniki, 54124 Thessaloniki, Greece; christop@cheng.auth.gr; 5 Laboratory of Pharmaceutical Technology, School of Pharmacy, Faculty of Health Sciences, Aristotle University of Thessaloniki, 54124 Thessaloniki, Greece; akapourag@pharm.auth.gr (A.K.); ipantazo@pharm.auth.gr (I.P.); pbarmp@pharm.auth.gr (P.B.)

**Keywords:** scaffold, bone graft, osseous defect, periodontitis, cone beam computed tomography, regenerative medicine, tissue engineering, 3D printing, fused deposition modeling, bioprinting

## Abstract

The treatment of osseous defects around teeth is a fundamental concern within the field of periodontology. Over the years, the method of grafting has been employed to treat bone defects, underscoring the necessity for custom-designed scaffolds that precisely match the anatomical intricacies of the bone cavity to be filled, preventing the formation of gaps that could allow the regeneration of soft tissues. In order to create such a patient-specific scaffold (bone graft), it is imperative to have a highly detailed 3D representation of the bone defect, so that the resulting scaffold aligns with the ideal anatomical characteristics of the bone defect. In this context, this article implements a workflow for designing 3D models out of patient-specific tissue defects, fabricated as scaffolds with 3D-printing technology and bioabsorbable materials, for the personalized treatment of periodontitis. The workflow is based on 3D modeling of the hard tissues around the periodontal defect (alveolar bone and teeth), scanned from patients with periodontitis. Specifically, cone beam computed tomography (CBCT) data were acquired from patients and were used for the reconstruction of the 3D model of the periodontal defect. The final step encompasses the 3D printing of these scaffolds, employing Fused Deposition Modeling (FDM) technology and 3D-bioprinting, with the aim of verifying the design accuracy of the developed methodοlogy. Unlike most existing 3D-printed scaffolds reported in the literature, which are either pre-designed or have a standard structure, this method leads to the creation of highly detailed patient-specific grafts. Greater accuracy and resolution in the macroarchitecture of the scaffolds were achieved during FDM printing compared to bioprinting, with the standard FDM printing profile identified as more suitable in terms of both time and precision. It is easy to follow and has been successfully employed to create 3D models of periodontal defects and 3D-printed scaffolds for three cases of patients, proving its applicability and efficiency in designing and fabricating personalized 3D-printed bone grafts using CBCT data.

## 1. Introduction

Several years after the creation of the first commercially accessible computed tomography (CT) scanner during the 1970s, a significant milestone occurred in 1998 when Mozzo et al. [1] developed the first cone beam computed tomography (CBCT) scanner, specifically designed for dental applications. This development marked a momentous breakthrough in the realm of medical imaging. Their study highlighted the enhanced three-dimensional (3D) visualization of dental structures and tissues with reduced radiation exposure compared to conventional CT scans. Subsequently, the CBCT technology underwent gradual integration into radiology and experienced widespread adoption within the dental field. It found extensive utility in various dental disciplines, including maxillofacial applications, implant design, assessing periodontal defects, supporting endodontic procedures, and aiding in orthodontics [2]. Over the past fifteen years, advancements in scanner-based 3D-modeling technologies [3], such as CBCT, have played a crucial role in aiding the diagnosis of certain diseases and the creation of personalized scaffolds (bone grafts) for medical purposes, particularly in the context of bone regeneration, by utilizing patient-specific anatomical data.

Accurately determining the 3D morphology of a periodontal defect is a challenge when relying solely on 2D digital X-rays. Consequently, the international literature has already proposed the use of CBCT for diagnosing periodontitis [4]. The key finding of the study conducted by AlJehani (2014) [4] suggests that CBCT imaging presents promising diagnostic applications for assessing periodontal diseases, offering detailed visualization of periodontal structures and facilitating precise treatment planning in dentistry. Numerous studies have demonstrated that CBCT scans are more effective than X-rays in identifying specific periodontal lesions [5,6,7]. Nevertheless, despite CBCT providing cross-sectional views in various orientations (sagittal, coronal, axial planes), it does not directly reveal the true morphology of the periodontal defect, unless a 3D model of the oral cavity is created using these data. Most studies primarily employ a simplistic thresholding segmentation method, leading to the creation of 3D models with high levels of noise and reduced accuracy [8,9,10], while other studies explore more complex partially automated segmentation techniques in dental CBCT scans using existing segmentation solutions [11,12,13,14]. Apart from the utilization of the available segmentation software, various automated methods have been employed for segmenting CBCT data in dental applications. Some of these methods include the utilization of morphological operators in conjunction with segmentation algorithms [15], the application of level-set methods [16], and the deployment of convolutional neural networks [17,18].

Addressing alveolar bone defects and restoring bone structures in patient treatment remains a challenging aspect of clinical practice in dentistry. Nevertheless, significant strides have been taken in incorporating scaffolding and 3D-printing technologies into oral and maxillofacial surgery, particularly in the context of periodontal and alveolar bone regeneration [19,20,21,22,23,24,25,26,27,28,29,30,31,32,33,34,35,36,37] and in numerous dental applications [38,39,40]. Studies reported in [19,20,21,22,23,24,25,26,27,28,29,30,31,32,33,34,35,36,37,38,39,40] demonstrate successful applications in periodontal and alveolar bone regeneration, with scaffolding technologies, biodegradable constructs, and antibacterial scaffolds showing promising outcomes. Furthermore, they showcase the potential of 3D-printed scaffolds in bone defect repair, apical periodontitis treatment, and coordinated periodontal-tissue engineering, underscoring the versatility and efficacy of 3D printing in periodontal regeneration. However, the scaffolds were either pre-designed or followed a standard structure, failing to mimic the morphological variations seen in actual cases of damage. Despite the recognition of CBCT technology’s potential for diagnosing periodontal diseases [41], CBCT has been underutilized in the development of patient-specific periodontal scaffolds. The pioneering clinical case of treating periodontitis in a patient using a CBCT-based 3D-printed scaffold was documented in [42]; in that study, encouraging outcomes were observed up to one year after the scaffold’s placement, including enhanced periodontal tissue regeneration and repair, reduction in periodontal pocket depths indicating improved periodontal health, favorable integration of the scaffold with surrounding tissues, and the maintenance of clinical stability and function over the follow-up period. Furthermore, the idea of generating scaffolds based on CBCT data has been explored in [43,44,45] for reconstructing alveolar bone defects. However, it is important to note that research into the 3D design of periodontitis scaffolds utilizing CBCT data remains relatively limited. The main gaps in the existing literature addressed by our study include the lack of a complete methodology for producing personalized 3D-printed scaffolds tailored to individual periodontal defects, the absence of technical specifics regarding the scaffold design processes using CBCT data, and the insufficient detail in representing fine structures of periodontal bone defects using existing processing algorithms.

In our prior studies, we introduced an approach for creating 3D models of hard tissues surrounding periodontal defects by using CBCT data [12] and designing 3D scaffolds (bone grafts) [13] that can be 3D-printed and placed for patient-specific periodontal defects for regeneration. The primary objective of this study is to refine and finalize our CBCT-based 3D-modeling pipeline presented in [12,13] and verify the accuracy of 3D-printed scaffolds for periodontal regeneration. Specifically, herein, we improve our methodology for CBCT-based generation of 3D models of alveolar bone and teeth surrounding periodontal defects and test our proposed approach for three new cases of patients. In addition, this study represents a significant advancement, with the 3D models crafted for the specific patients undergoing 3D printing using Fused Deposition Modeling (FDM) and 3D-bioprinting technology. This step is undertaken to validate the precision of the designed models and to evaluate the final 3D scaffolds in terms of both their quality and their effectiveness in addressing patients’ periodontal defects.

## 2. Materials and Methods

### 2.1. 3D Modeling of Periodontal Defects

In our previous study [12], we conducted an assessment of segmentation and 3D-modeling procedures utilizing dental CBCT data. This involved conducting various comparisons of 3D models of the hard tissues within the oral cavity of patients with periodontitis, generated using different techniques. As a result of this research, we defined a preferred methodology specifically tailored for 3D modeling of the teeth and alveolar bone in areas where periodontal defects are detected in patients with periodontitis. After some further experiments using the methodology proposed in [12], in the present study we utilize a variation of the latter, regarding the number of segments defined in the segmentation process of the CBCT dataset. Specifically, in the originally proposed methodology [12], we defined three segments, namely, teeth, alveolar bone, and “other”, encompassing all the other regions of the oral system visible in the cropped CBCT images. In order to further reduce the computational time for the segmentation and the 3D-modeling processes, and considering that there is no need to separate the alveolar bone and the teeth into distinct 3D models, as described in our previous study [12], only one segment has been defined for hard tissues (teeth and alveolar bone) around the periodontal lesion. This refined methodology can be summarized as outlined in the following:

1. Defining the area of interest by cropping the relevant volume within the CBCT dataset.
2. Defining two segments: hard tissues (for the teeth and alveolar bone) and “other”, encompassing all the other regions of the oral system visible in the cropped CBCT images.
3. Identifying representative samples for these segments for a limited subset of CBCT images (e.g., thirty images evenly distributed across ten per reference plane), using a mix of automated (thresholding) and manual approaches.
4. Initializing the “Grow from seeds” technique [46].
5. Repeatedly correcting and updating the output of the “Grow from seeds” method till the visual result meets the operator’s satisfaction, ensuring that the segment of hard tissues exclusively contains teeth and alveolar bone, with the segment “other” devoid of any periodontal defect regions. During this stage, it is advisable to overlook minor errors or noise that can be quickly and easily corrected using 3D-editing software, in order to expedite the overall process.
6. Converting the segmentation result for the segment of hard tissues into a 3D model (mesh) in the STL format.
7. Editing the 3D model of hard tissues, which may include steps such as converting the 3D mesh into a point cloud, editing the point cloud and constructing a 3D mesh (surface) based on the edited point cloud, which is then saved in the STL format.

Aside from the final stage of the described procedure, which can be achieved using dedicated 3D-editing software such as Geomagic Wrap (Artec3D, Luxemburg) [47], all the preceding steps can be executed via medical imaging software, like the open-source software 3D Slicer [48].

### 2.2. 3D Design of Patient-Specific Scaffolds

In our latest study [13], we presented two types of methodology for designing 3D periodontal scaffolds. The initial method pertains to designing periodontal defect customized block grafts; the second one involves creating extraction socket preservation customized grafts. For the sake of completeness, in this section we provide a summary of these types of methodologies.

The design of periodontal defect customized block grafts includes the steps summarized in the following:

1. Stage A: The design of an initial approximation of the scaffold internal surface (i.e., the surface of the scaffold that touches the tooth (laterally) and the alveolar bone (at the bottom of the scaffold if it is a mandibular scaffold or on the upper part of the scaffold, if it is a maxillary scaffold), using the 3D model of the periodontal defect, implementing operations like cutting, hole filling, noise reduction, 3D surface smoothing and inversion of the normal vectors).
2. Stage B: The design of the scaffold outer surface (i.e., its surface that does not come into contact with the patient’s hard tissues), using the aforementioned model, through operations like copying parts of the 3D surface, cutting, moving, application of a rotation transformation, and, possibly, translation and scaling, smoothing, noise reduction, erasing of parts of the surface not well reconstructed, hole filing, merging, and smoothing.
3. Stage C: The design of an ultimate representation of the scaffold’s internal surface, by cropping the 3D model produced in stage A using the result of Stage B.
4. Stage D: The design of the final scaffold through the creation of the missing surfaces and final processing.

The design of extraction socket preservation customized grafts includes the steps summarized in the following.

1. Stage A: The design of the internal surface of the scaffold (same as Stage A of the process of designing a periodontal defect customized block graft).
2. Stage B: The design of the final scaffold through a hole-filling methodology followed by further processing.

All these stages for the design of patient-specific 3D bone grafts may be conducted using a 3D-model-editing software, like Geomagic Wrap [47].

### 2.3. CBCT Scanner and Software Utilized

The research protocol using humans in the frame of the 3D-PerioDontis project, has been approved by the Ethics and Research Integrity Committee of the Aristotle University of Thessaloniki, Greece (#212202/2021).

Patients with severe periodontitis receiving medical treatment were informed about the scope of the project. Given that they meet the admissions criteria (several participation and exclusion criteria were set by the research team), those that wanted to voluntarily participate in the project had to sign a consent form. In that case, their medical history and personal data were recorded and bone lesions were depicted with the use of CBCT—no therapy related to the project was applied. Data are coded for future use so that no correlation can be further realized (anonymization of data) and saved for 30 months, while access is available only to the principal investigator of the project. Any volunteer could stop the study at any time and request his/her data to be deleted.

The study analyzed anonymous DICOM (Digital Imaging and Communications in Medicine) data (as above) from mandibular CBCT scans of three patients diagnosed with periodontitis. Patient-specific details on diagnostic or treatment parameters are not available. The scanner used was the NEWTOM VGI evo CBCT machine (CEFLA, Imola, BO, Italy). A high-resolution-imaging protocol with a 150-micron voxel size was employed; exposure settings of 110 KV and 109.2 mAs were used, and a standard 100 mm × 100 mm field of view was utilized. The scanning procedure adhered to the recommendations of the manufacturer.

Regarding the software used, the segmentation of the CBCT images and initial 3D model creation of the hard tissues of the oral system for the three patients in the area of their periodontal defects were performed using an open-source medical-image-editing software, namely, 3D Slicer, version 4.11.20210226 [48]. The commercial software Geomagic Wrap 2017 [47] was employed to process the aforementioned 3D models for the three patients, generate the final 3D models of their hard tissues in the area of their periodontal defects, and design the 3D scaffolds.

### 2.4. FDM 3D Printing

A Flash Forge Creator Pro (Zhejiang Flashforge 3D Technology Co., Jinhua, Zhejiang, China) A Fused Deposition Modeling (FDM) 3D printer was used to print the designed scaffolds. A single extrusion head was utilized containing a single commercial polylactic acid (PLA) filament with an external diameter of 1.75 mm (Flashforge PLA Pro, A FlashPrint, Zhejiang Flashforge, Zhejiang, China). The platform temperature was set at 45 °C, and the printing temperature was set at 200 °C for all samples. In order to facilitate the printing of the scaffolds, the STL files were processed using the Flash Print software version 4.3.0 (FlashPrint, Zhejiang Flashforge, China). For the printing of the scaffolds, three different printing profiles were tested, namely, fast, fine, and standard. The parameters for each profile are defined in Table 1. The selected fill pattern, which represents the deposition procedure for the 3D-scaffold printing, was hexagonal.

### 2.5. 3D Bioprinting

A BIO-X 3D printer (Cellink, Gothenburg, Sweden) was used to print the designed scaffolds. Two different materials were used: (a) poly(ε-caprolactone) (PCL) 3D filament with an external diameter of 1.75 mm and a density of 1.145 g/cm^3^ (3D4MAKERS, Haarlem, The Netherlands) and (b) polylactic acid (PLA) 3D filament with an external diameter of 1.75 mm and a density of 1.25 ± 0.05 g/cm^3^ (Flashforge PLA Pro, A FlashPrint, Zhejiang Flashforge, China).

For the printing process, both materials were chopped into pellets to fit in the cartridge of the BIO-X 3D printer. For both materials, approximately 20 g of pellets were placed into the cartridge and preheated at 100 °C for PCL and 180 °C for PLA, for at least 20 min. The nozzle tip used for the printing process had a diameter of 400 μm. The bed temperature was maintained stably at 25 °C. Printing settings, such as temperature, speed, and extrusion pressure, were all variated depending on the design features of each scaffold design. The settings are as shown in Table 2.

## 3. Results

In this section, the results of the methodology for 3D-modeling periodontal defects, designing 3D models of scaffolds and deriving 3D-printed scaffolds, discussed in Section 2, are presented. Specifically, we generated the 3D models of periodontal defects for three cases of patients, using CBCT data, and we designed and printed the 3D models of six scaffolds (two scaffolds per patient).

### 3.1. Patient 1

#### 3.1.1. 3D Model of Periodontal Defect

**Figure 1:**
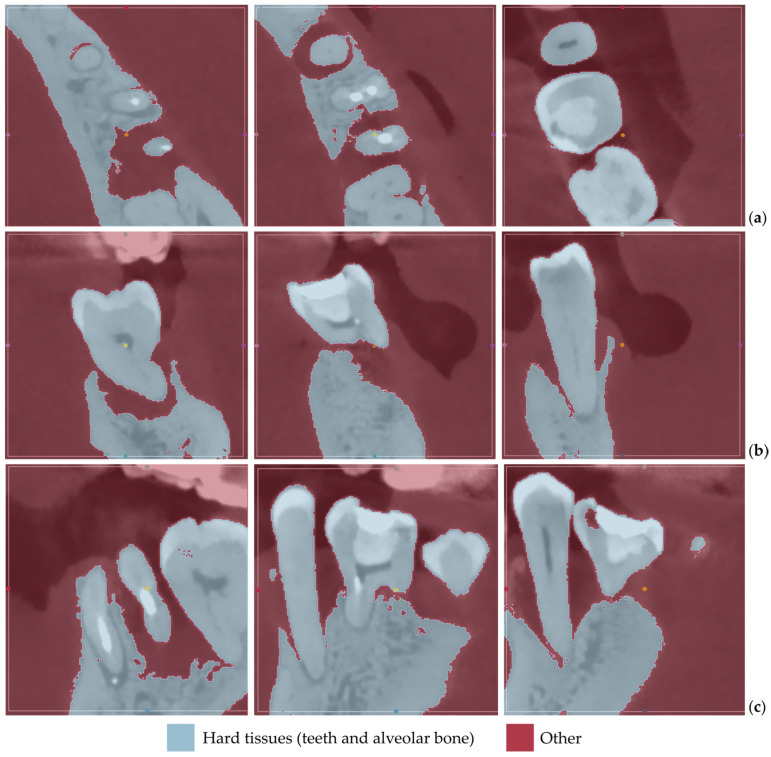
“Grow from seeds” segmentation results for Patient 1, for a subset of CBCT images (after cropping to include only the area of interest) in the axial plane (**a**), the coronal plane (**b**), and the sagittal plane (**c**).

Figure 1 displays the segmentation outcomes obtained from the “Grow from seeds” method applied to a subset of tomographic images derived from Patient 1′s CBCT scan, in the axial, coronal, and sagittal planes.

From the images shown in Figure 1, the effectiveness of the “Grow from seeds” method in segmenting the dataset becomes evident. In step 5, when examining the method’s outcome and refining the samples iteratively (see Section 2.1), those errors that can be easily fixed through processing the created 3D model were not manually adjusted.

**Figure 2:**
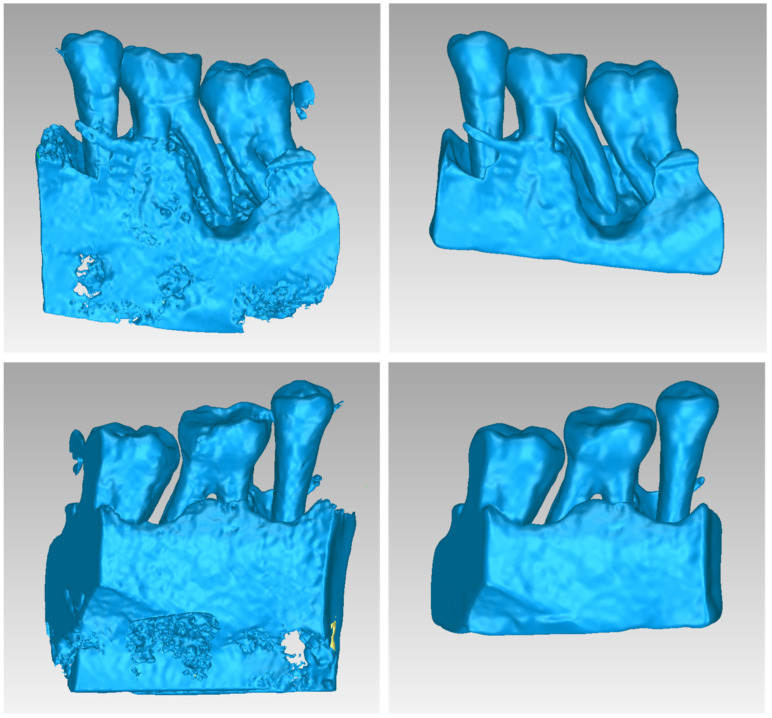
3D model of the hard tissues (teeth and alveolar bone) of Patient 1 in the area of the periodontal defect before processing, as resulted from the segmentation process (**left**), and after processing (**right**), from two different viewpoints.

Figure 2 illustrates the 3D model of the hard tissues in the same 3D space from two distinct perspectives. The left image portrays the segmentation-derived 3D model before any processing, while the right image presents the same model after undergoing processing. Hence, Figure 2 clearly highlights the difference between the initial 3D model generated using segmentation and the refined final 3D model after processing. Thus, 3D-model processing, as proposed in the methodology summarized in Section 2.1, is deemed necessary.

**Figure 3:**
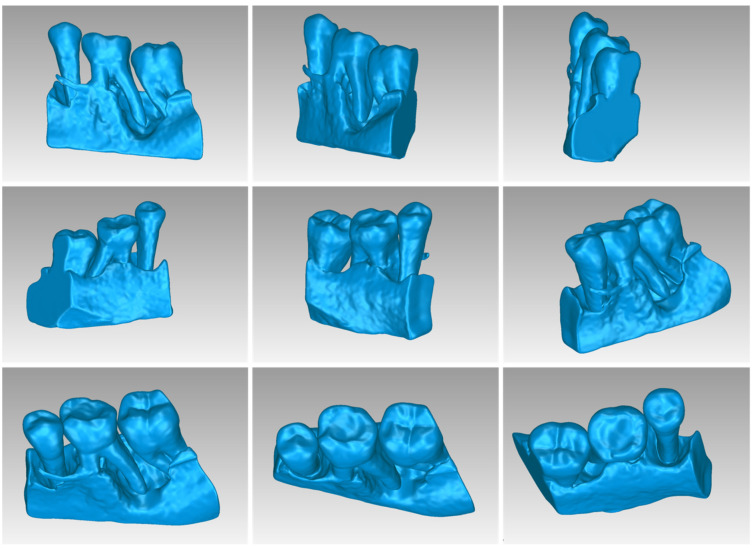
Final 3D model of hard tissues surrounding the periodontal defect of Patient 1, as seen from nine different viewpoints.

Table 3 displays the number of triangles and the size of the 3D model of the hard tissues around the periodontal defect (teeth and alveolar bone) of Patient 1 before and after processing. The final 3D model of the hard tissues (after processing) is shown in Figure 3.

#### 3.1.2. 3D Models of Scaffolds

**Figure 4:**
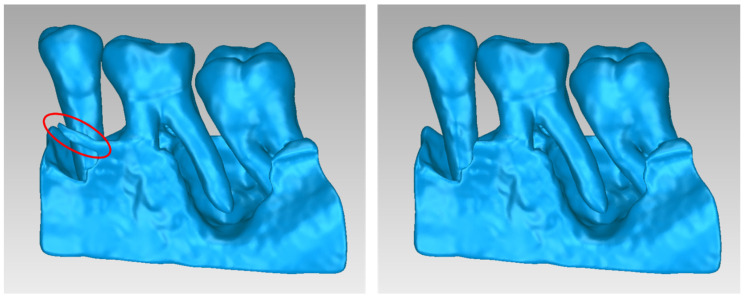
Final 3D model of the hard tissues of the mandible of Patient 1 where the periodontal defect is focused before the surgery (**left**) and after the surgery (**right**) that is required for placing the scaffolds for periodontal regeneration. The part of the alveolar bone that needs to be surgically cut for placement of the scaffolds is indicated with red ellipse (**left**).

**Figure 5:**
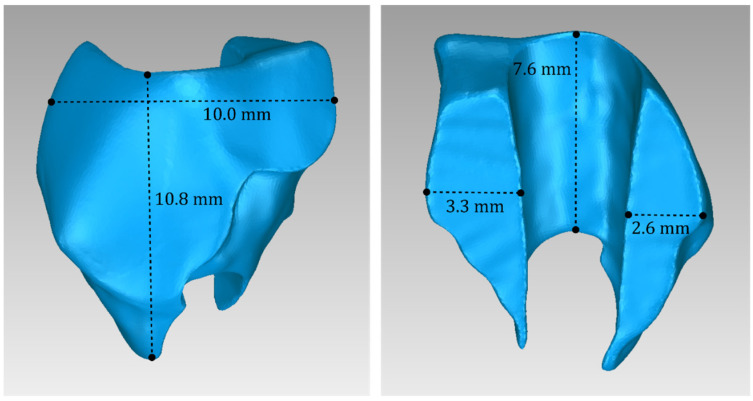
Views of the 3D model of scaffold 1 designed for Patient 1, and illustration of selected dimensions thereof.

**Figure 6:**
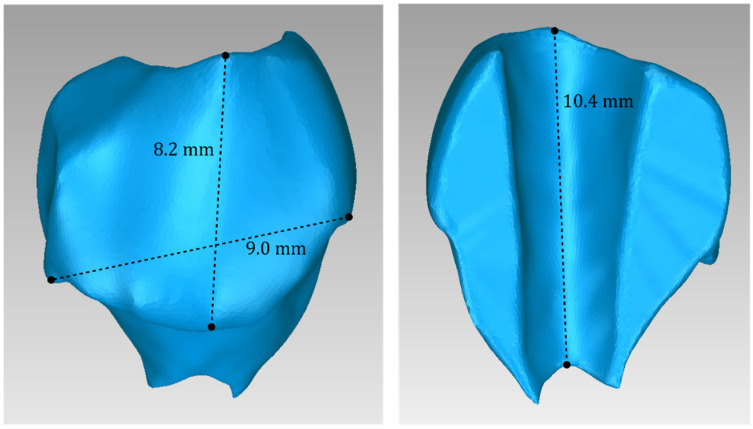
Views of the 3D model of scaffold 2 designed for Patient 1, and illustration of selected dimensions thereof.

**Figure 7:**
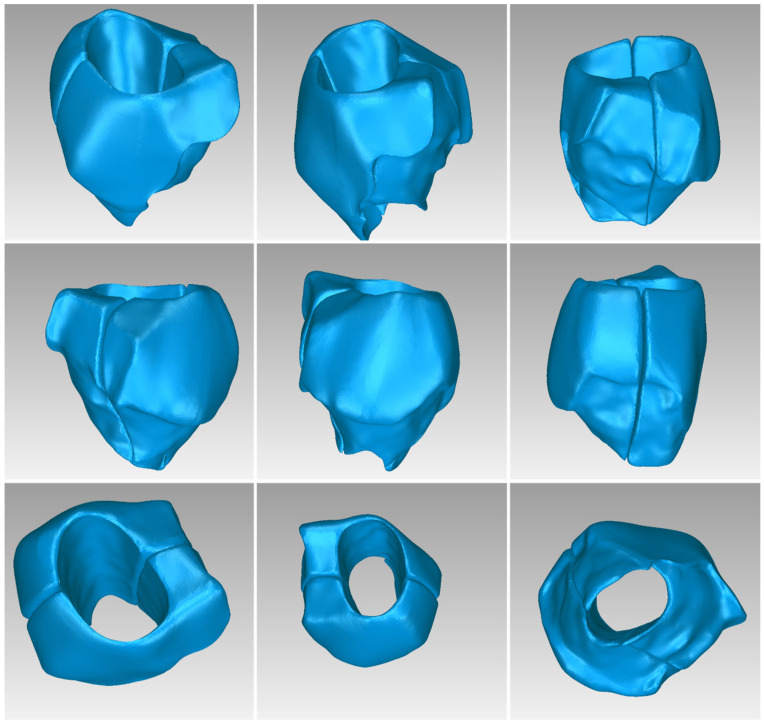
Views of the 3D models of scaffolds 1 and 2 of Patient 1.

**Figure 8:**
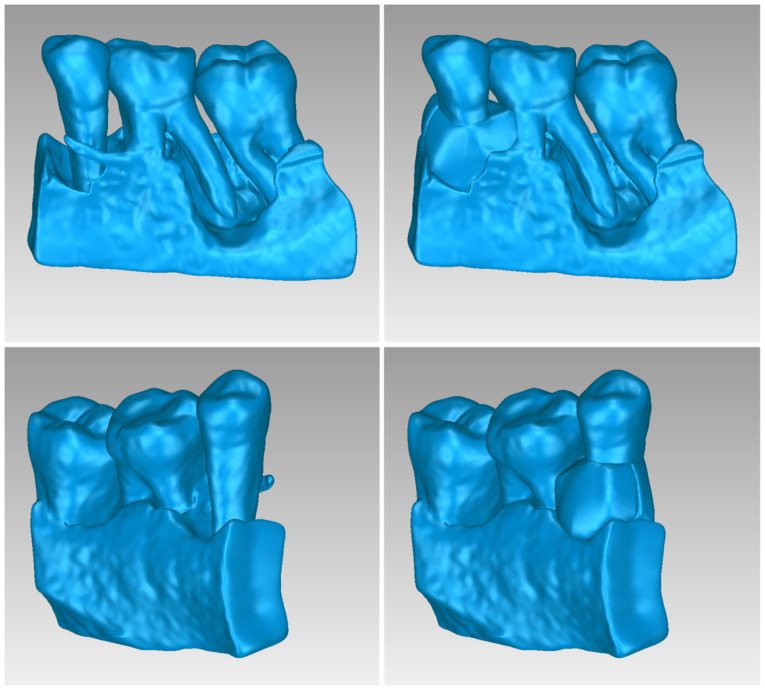
Left: 3D model of the periodontal defect of Patient 1. Right: 3D model of the periodontal defect of Patient 1 and 3D models of scaffolds.

**Figure 9:**
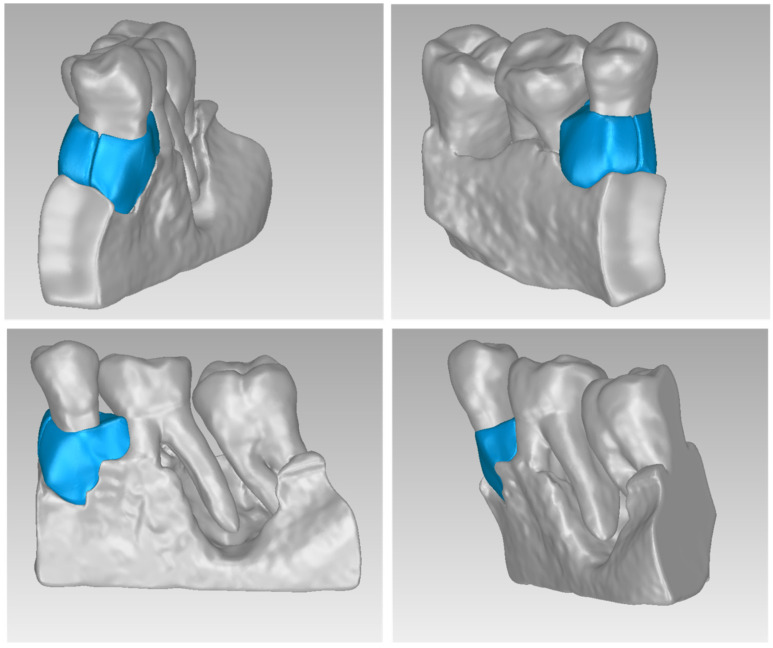
3D model of the periodontal defect of Patient 1 (grey), and 3D models of the generated scaffolds (blue), in the same 3D space from various perspectives.

For Patient 1, two 3D models of scaffolds were designed, based on the 3D model of the hard tissues where the periodontal lesion is focused. In order to place the scaffolds on this patient, a surgery is needed to remove part of the patient’s alveolar bone. The 3D model of the hard tissues of Patient 1 before the said surgery (left) and after the surgery (right), which is required for placing the scaffolds, is shown in Figure 4. The red-color ellipse in Figure 4 (left) indicates the part of the alveolar bone which is required to be surgically cut, for the placement of the scaffolds. The 3D model of the two scaffolds of Patient 1, which were generated following the approach presented in Section 2.2, and their dimensions are illustrated in Figure 5 and Figure 6. The volume of scaffold 1 of Patient 1 is 169.75 mm^3^. It consists of 38,404 triangles and has a size of 1.8 MB. The volume of scaffold 2 of Patient 1 is 202.14 mm^3^, consisting of 27,720 triangles and with a size of 1.3 MB. Views of the 3D models of the scaffolds generated for Patient 1 are presented in Figure 7. Figure 8 shows the 3D model of the periodontal defect without the designed scaffolds (left) as well as with the 3D models of the two designed scaffolds (right). Figure 9 showcases the 3D representation of Patient 1′s periodontal defect alongside the 3D models of the two designed scaffolds, all positioned within the same 3D space but viewed from various perspectives.

#### 3.1.3. 3D-Printed Scaffolds

**Figure 10:**
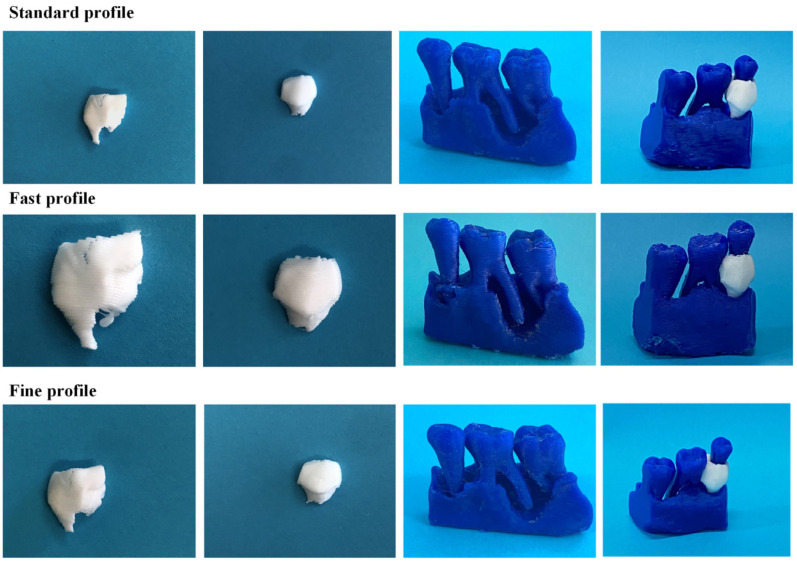
Images of the FDM 3D-printed scaffolds for Patient 1, produced using the three different printing profiles (from left to right: scaffold 1, scaffold 2, alveolar bone, and scaffolds applied to the periodontal defect).

Figure 10 illustrates the 3D-printed scaffolds pertaining to Patient 1, produced utilizing the FDM 3D printer and employing the three distinct printing profiles. Upon comparative analyses of the scaffolds, those printed with the fast profile displayed diminished precision and quality. Additionally, when introduced into the periodontal defect, these scaffolds demonstrated a less-optimal fit compared to those printed with the standard and fine profiles.

Table 4 presents the printing time and the material consumption necessary for the fabrication of the scaffolds of Patient 1 across each respective printing profile. From the obtained results, it is evident that the fine printing profile requires a significantly higher printing time compared to the other two profiles, as well as a greater amount of material. Conversely, the fast profile exhibits the least demands in terms of printing time.

**Figure 11:**
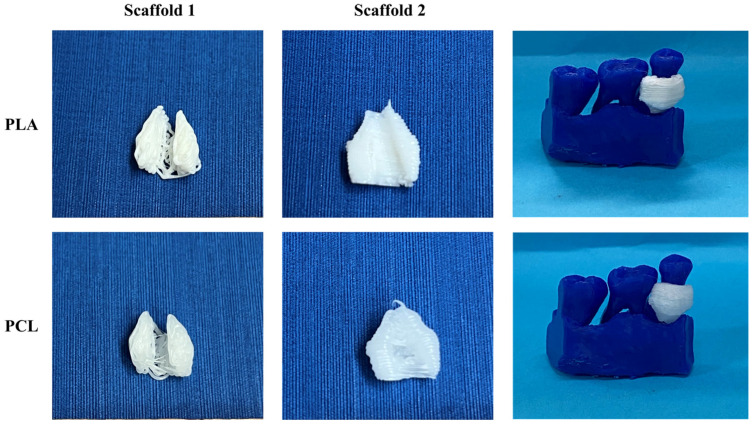
Images of the 3D-bioprinted scaffolds for Patient 1, produced using two different materials (from left to right: scaffold 1, scaffold 2, alveolar bone with the scaffolds applied to the periodontal defect).

Figure 11 illustrates the 3D-printed scaffolds pertaining to Patient 1, produced with bioprinting technology using two different materials. The PCL scaffolds exhibited better printing resolution and a smoother surface than that of the PLA scaffolds. Additionally, when introduced into the periodontal defect, PCL scaffolds demonstrated a better fit around the tooth, compared to those printed with PLA material.

### 3.2. Patient 2

#### 3.2.1. 3D Model of Periodontal Defect

**Figure 12:**
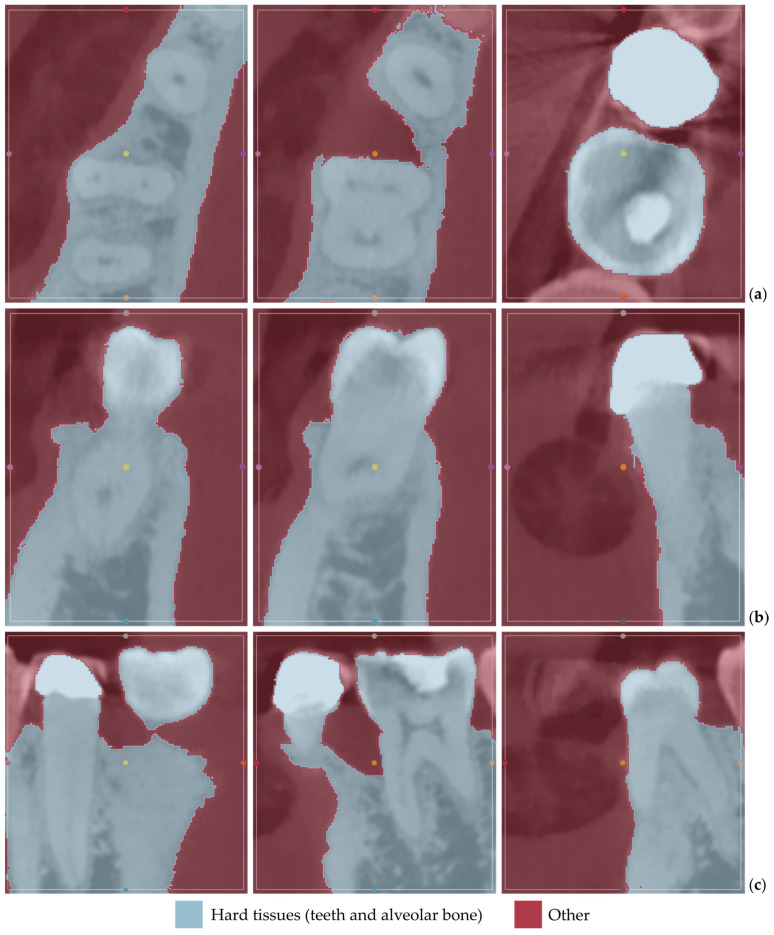
“Grow from seeds” segmentation results for Patient 2, for a subset of CBCT images (after cropping to include only the region of interest) in the axial plane (**a**), the coronal plane (**b**), and the sagittal plane (**c**).

Figure 12 displays the segmentation outcomes obtained from the “Grow from seeds” method applied to a subset of tomographic images derived from Patient 2′s CBCT scan, in the axial, coronal, and sagittal planes.

From the images shown in Figure 12, the effectiveness of the “Grow from seeds” method in segmenting the dataset becomes evident, once more. Similar to the case of Patient 1, in step 5, when examining the method’s outcome and refining the samples iteratively (see Section 2.1), those errors that can be easily fixed through processing the created 3D model were not manually adjusted.

**Figure 13:**
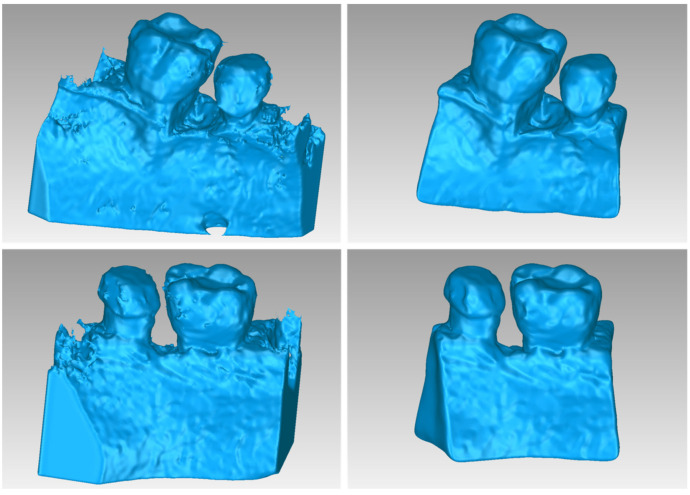
3D model of the hard tissues (teeth and alveolar bone) of Patient 2 in the area of the periodontal defect before processing, as resulted from the segmentation process (**left**), and after processing (**right**), from two different viewpoints.

Figure 13 illustrates the 3D model of the hard tissues of Patient 2 in the same 3D space from two distinct perspectives. The left image portrays the segmentation-derived 3D model before any processing, while the right image presents the same model after undergoing processing. From this Figure, the necessity of processing the 3D model resulted from the segmentation is noticeable.

**Figure 14:**
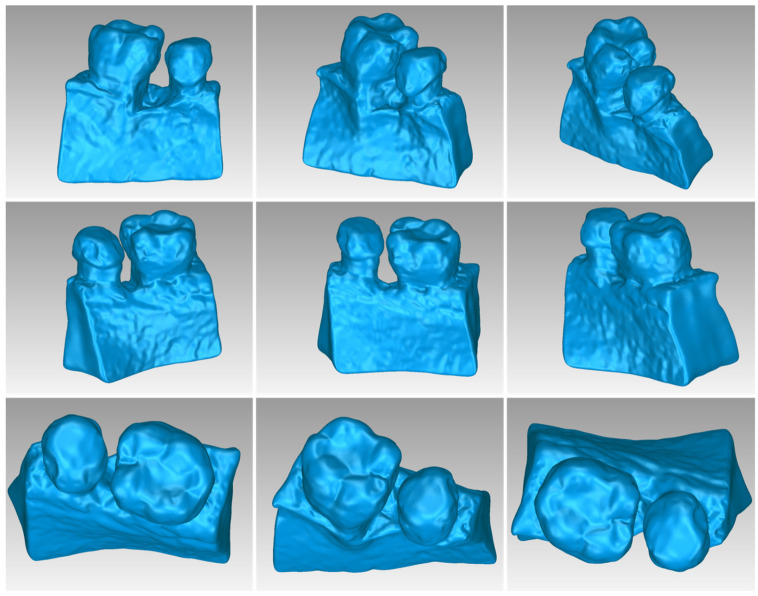
Final 3D model of hard tissues in the area of the periodontal defect of Patient 2, as seen from nine different viewpoints.

Table 5 shows the number of triangles and the size of the 3D model of the alveolar bone and teeth surrounding the periodontal defect of Patient 2 before and after processing. The final 3D model of the hard tissues (after processing) is shown in Figure 14.

#### 3.2.2. 3D Models of Scaffolds

**Figure 15:**
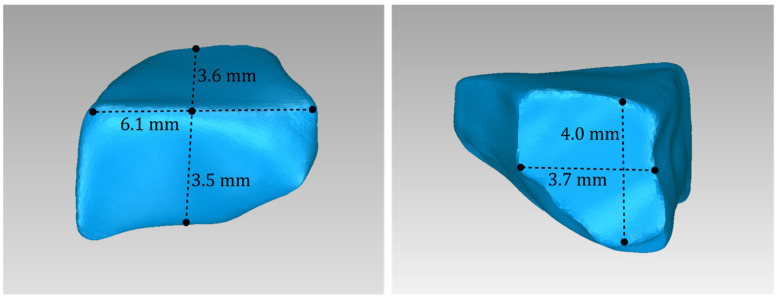
Views of the 3D model of scaffold 1 designed for Patient 2, and illustration of selected dimensions thereof.

**Figure 16:**
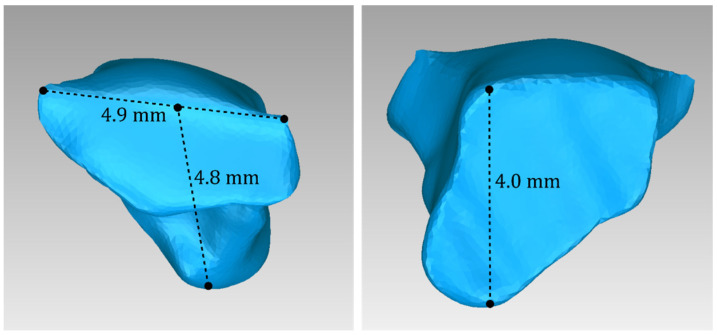
Views of the 3D model of scaffold 2 designed for Patient 2, and illustration of selected dimensions thereof.

**Figure 17:**
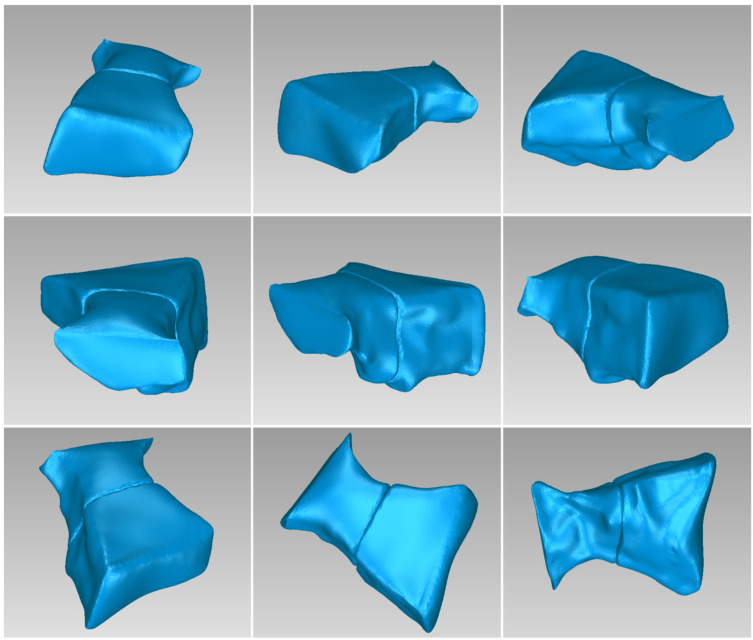
Views of the 3D models of scaffolds 1 and 2 of Patient 2.

**Figure 18:**
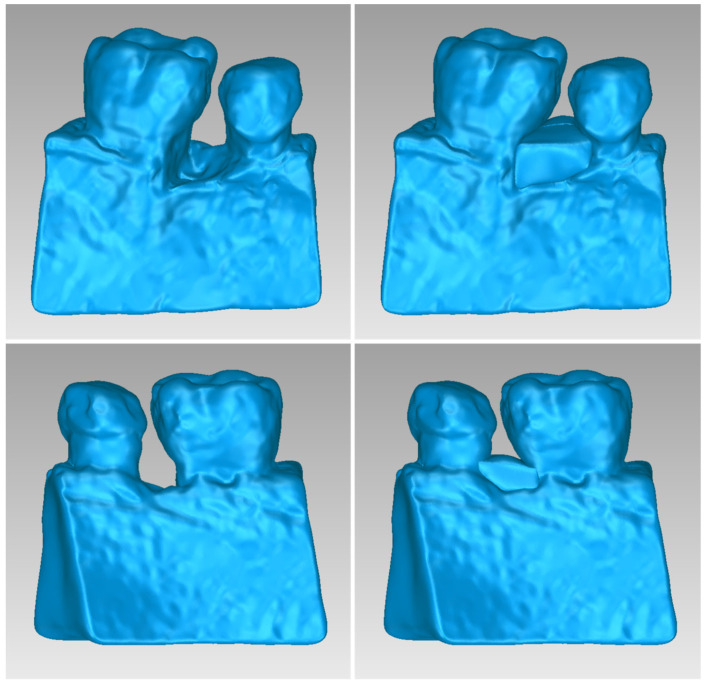
Left: 3D model of the periodontal defect of Patient 2. Right: 3D model of the periodontal defect of Patient 2 and 3D models of scaffolds.

**Figure 19:**
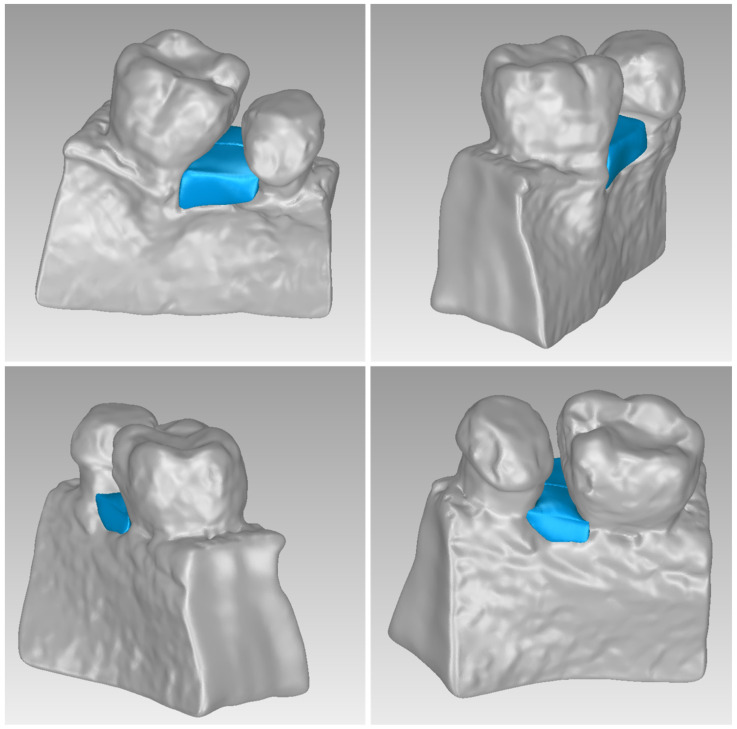
3D model of the periodontal defect of Patient 2 (grey), and 3D models of the generated scaffolds (blue), in the same 3D space from various perspectives.

Two 3D models of scaffolds were designed for Patient 2, using the 3D model of the hard tissues around the periodontal defect. The 3D models of the two scaffolds, generated according to the methodology discussed in Section 2.2, and their dimensions, are illustrated in Figure 15 and Figure 16. The volume of scaffold 1 of Patient 2 is 73.50 mm^3^, consisting of 14,796 triangles and corresponding to a size of 0.7 MB. The volume of scaffold 2 of Patient 2 is 31.45 mm^3^, consisting of 9596 triangles and having a size of 0.5 MB. Views of the 3D models of the scaffolds designed for Patient 2 are presented in Figure 17. Figure 18 shows the 3D model of the periodontal defect without the scaffolds that have been designed (left) as well as with the 3D models of both scaffolds (right). Figure 19 illustrates the 3D reconstruction of Patient 2′s periodontal defect alongside the 3D representations of the two scaffolds, all placed within the same 3D space but observed from varying perspectives.

#### 3.2.3. 3D-Printed Scaffolds

**Figure 20:**
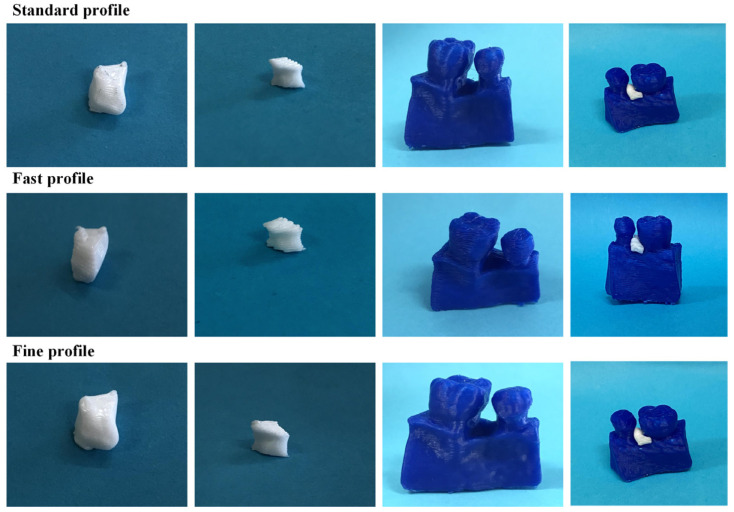
Images of the FDM 3D-printed scaffolds for Patient 2, produced using the three different printing profiles (from left to right: scaffold 1, scaffold 2, alveolar bone, and scaffolds applied to the periodontal defect).

As in the case of Patient 1, the 3D printing of the scaffolds for Patient 2 was carried out using the FDM printer. The printed scaffolds, utilizing the three printing profiles described in Section 2.4, are depicted in Figure 20. Additionally, Table 6 provides the printing time and material consumption of the printing process. Based on the obtained results, a greater precision and quality in 3D-printed scaffolds were achieved with the fine profile. However, it had the highest requirements in both printing time and material consumption. In contrast, the fast profile produced less-precise scaffolds but did so in a shorter time, while the standard profile balanced these aspects, offering intermediate printing times and excellent quality, with scaffolds that precisely fit the defect.

**Figure 21:**
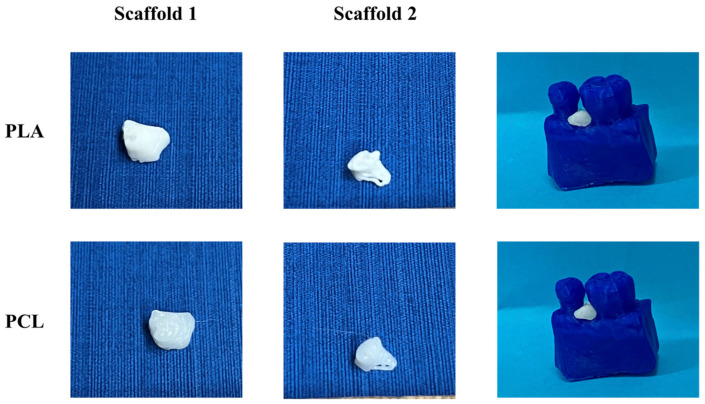
Images of the 3D-bioprinted scaffolds for Patient 2, produced using two different materials (from left to right: scaffold 1, scaffold 2, alveolar bone with scaffolds applied to the periodontal defect).

Figure 21 illustrates the 3D-printed scaffolds pertaining to Patient 2, produced with bioprinting technology with two different materials. Upon comparative analyses of the scaffolds, the PCL scaffolds exhibited a smoother surface and better macroarchitecture compared to scaffolds made out of PLA. However, when introduced into the periodontal defect, both scaffolds did not exhibit a good fit in-between the teeth.

### 3.3. Patient 3

#### 3.3.1. 3D Model of Periodontal Defect

**Figure 22:**
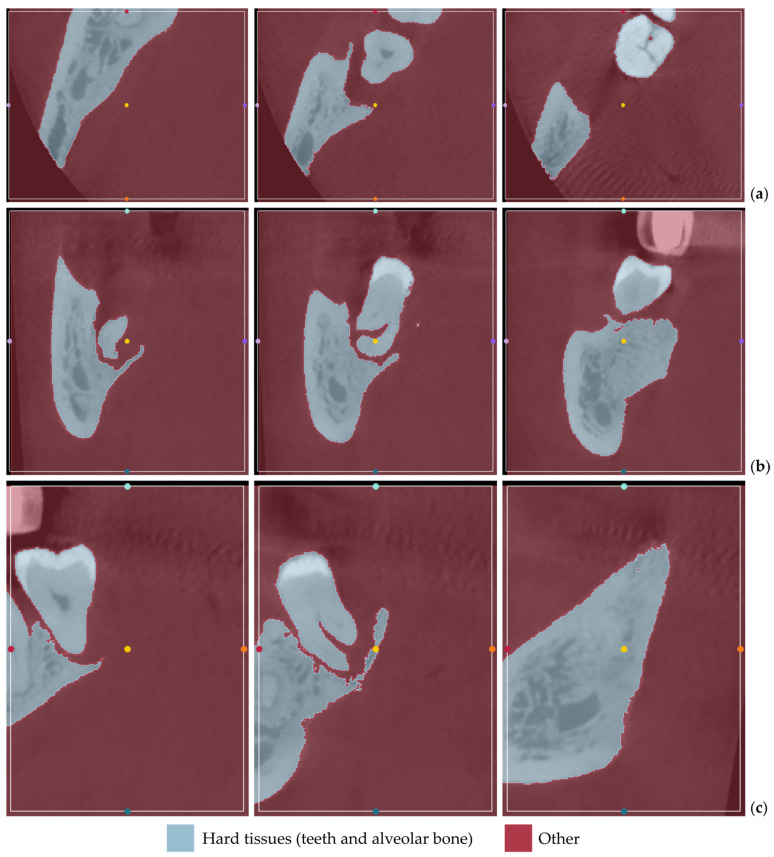
“Grow from seeds” segmentation results for Patient 3, for a subset of CBCT images (after cropping to include only the region of interest) in the axial plane (**a**), the coronal plane (**b**), and the sagittal plane (**c**).

Figure 22 displays the segmentation outcomes obtained from the “Grow from seeds” method applied to a subset of tomographic images derived from Patient 3′s CBCT scan.

As seen in Figure 22, the “Grow from seeds” method has yielded satisfactory segmentation results. Similar to the cases of Patient 1 and Patient 2, during step 5 of the 3D-modeling procedure described in Section 2.1, those errors that can be easily fixed through processing of the created 3D model have not been manually corrected in the segmentation results of Patient 3.

**Figure 23:**
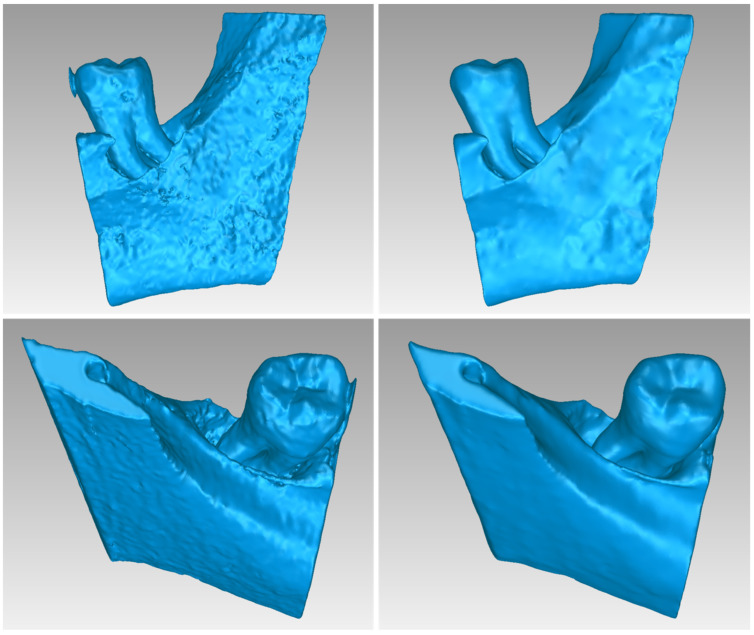
3D model of the hard tissues (teeth and alveolar bone) of Patient 3 in the area of the periodontal defect before processing, as resulted from the segmentation process (**left**), and after processing (**right**), from two different viewpoints.

Figure 23 illustrates the 3D model of the hard tissues of Patient 3 in the same 3D space from two distinct perspectives. The left image portrays the segmentation-derived 3D model before any processing, while the right image presents the same model after undergoing processing. This figure highlights the necessity of processing the segmentation-based 3D model.

**Figure 24:**
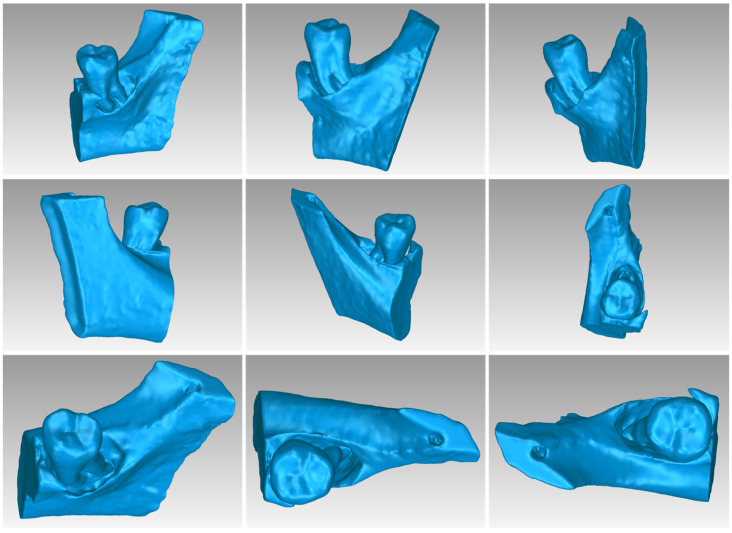
Final 3D model of hard tissues in the area of the periodontal defect of Patient 3, as seen from nine different viewpoints.

Table 7 shows the number of triangles and the size of the 3D model of the hard tis-sues surrounding the periodontal defect (teeth and alveolar bone) of Patient 3 before and after processing. The final 3D model of the hard tissues (after processing) is shown in Figure 24.

#### 3.3.2. 3D Models of Scaffolds

**Figure 25:**
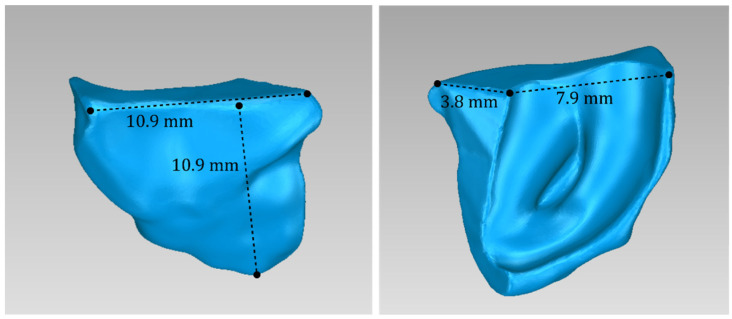
Views of the 3D model of scaffold 1 designed for Patient 3, and illustration of selected dimensions thereof.

**Figure 26:**
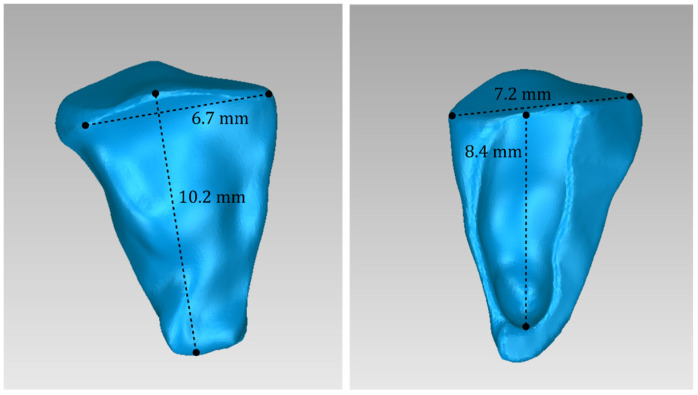
Views of the 3D model of scaffold 2 designed for Patient 3, and illustration of selected dimensions thereof.

**Figure 27:**
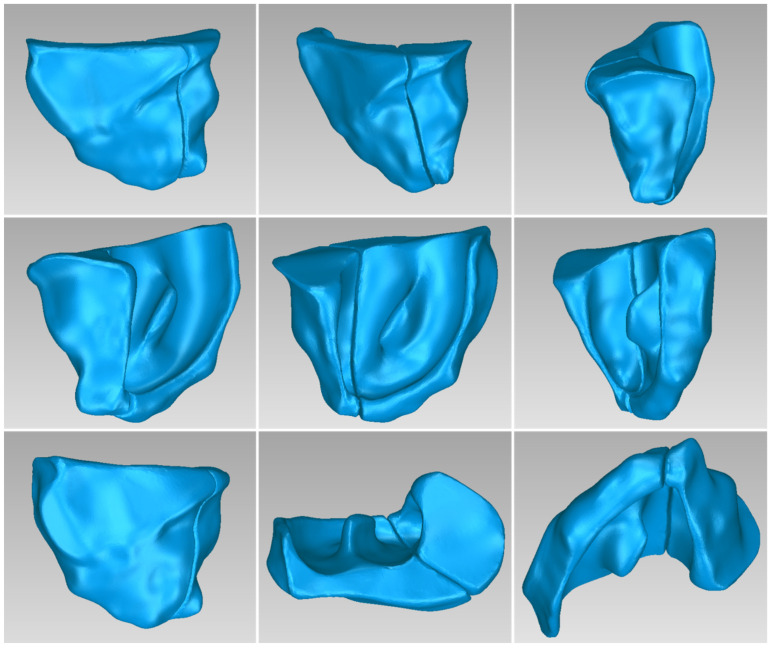
Views of the 3D models of scaffolds 1 and 2 of Patient 3.

**Figure 28:**
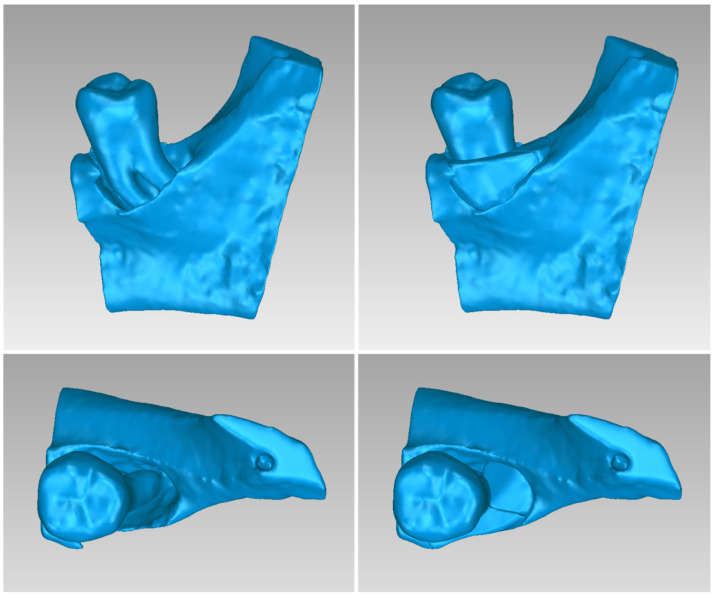
(**Left**): 3D model of the periodontal defect of Patient 3. (**Right**): 3D model of the periodontal defect of Patient 3 and 3D models of scaffolds.

**Figure 29:**
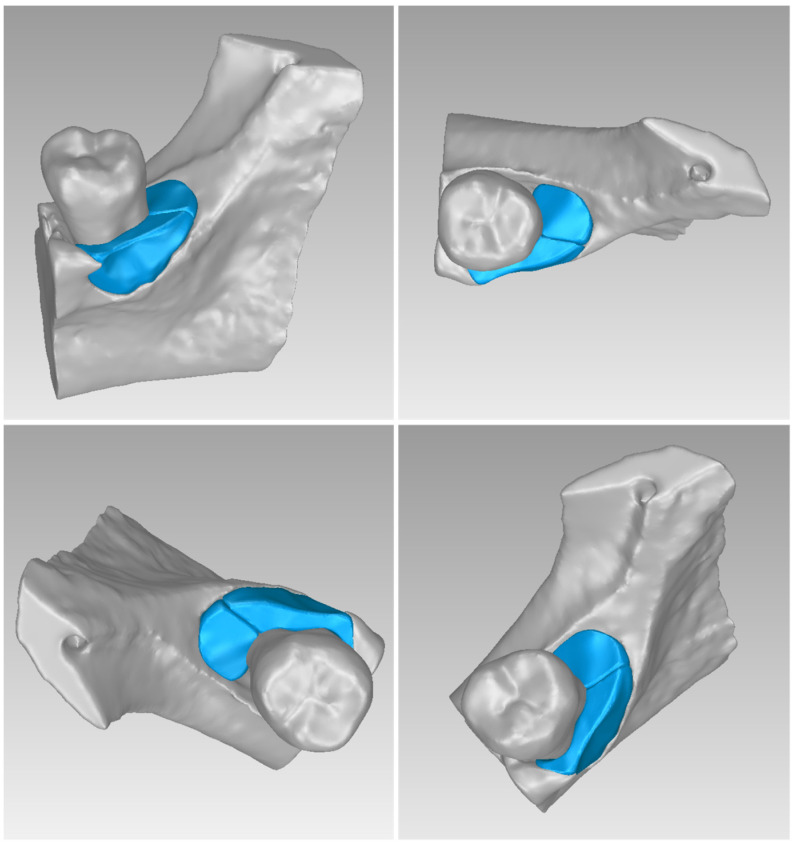
3D model of the periodontal defect of Patient 3 (grey) and 3D models of the generated scaffolds (blue), in the same 3D space from various perspectives.

Two 3D models of scaffolds were designed for Patient 3, using the 3D representation of the hard tissues surrounding the periodontal defect. The 3D models of the two scaffolds, that were generated for Patient 3 following the approach discussed in Section 2.2, and their dimensions, are illustrated in Figure 25 and Figure 26. The volume of scaffold 1 of Patient 3 is 217.18 mm^3^, consisting of 41,648 triangles and corresponding to a size of 2.0 MB. The volume of scaffold 2 of Patient 3 is 113.40 mm^3^, consisting of 25,290 triangles and with a size of 1.2 MB. Views of the 3D models of the scaffolds designed for Patient 3 are presented in Figure 27. Figure 28 shows the 3D model of the periodontal defect of Patient 3 without the designed scaffolds (left) as well as with the 3D models of both designed scaffolds (right). Figure 29 showcases the 3D representation of Patient 3′s periodontal defect alongside the 3D models of the two designed scaffolds, all positioned within the same 3D space but viewed from various perspectives.

#### 3.3.3. 3D-Printed Scaffolds

**Figure 30:**
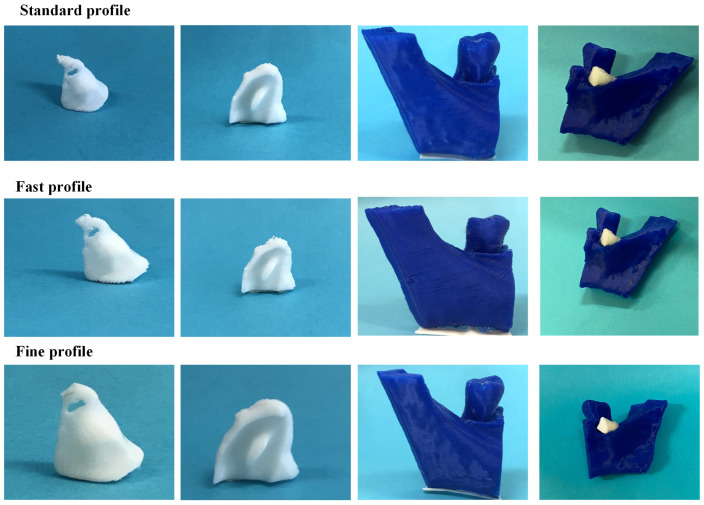
Images of the FDM 3D-printed scaffolds for Patient 3, produced using the three different printing profiles (left to right: scaffold 1, scaffold 2, the alveolar bone, and the scaffolds applied to the periodontal defect.).

Figure 30 shows the scaffolds produced with the FDM 3D printer for Patient 3.

In the fabrication of scaffolds for this patient, there were no discernible distinctions when compared to the scaffolds printed for the two preceding patients. Specifically, considering the printing time and material requirements outlined in Table 8, the standard printing profile proved to be the optimal choice, striking a balance between quality, printing time, and material consumption.

**Figure 31:**
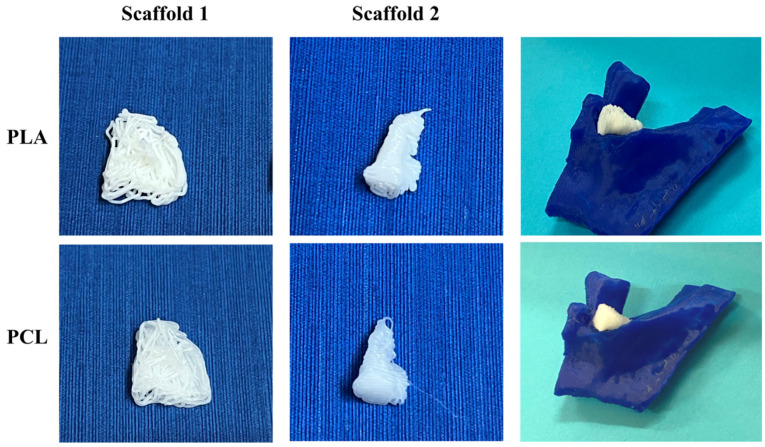
Images of the 3D-bioprinted scaffolds for Patient 3, produced using two different materials (left to right: scaffold 1, scaffold 2, the alveolar bone with the scaffolds applied to the periodontal defect.).

In Figure 31, both scaffolds produced with the bioprinter for Patient 3 failed at achieving high resolution for their macroarchitecture. This can be attributed to the fact that these scaffolds had the most demanding curvatures architectures. Nevertheless, both scaffolds seemed to fill the void of the defect, but without filling the gap around the tooth.

## 4. Discussion

The treatment of bone defects surrounding teeth is a fundamental concern within the field of periodontology. These defects arise from prolonged inflammation of the gum and periodontal tissues caused by various bacteria, resulting in bone loss around teeth and weakened tooth support. The primary objective of periodontal treatment is to eliminate underlying causes and, when feasible, facilitate the regeneration of bone to support tooth structures. Over the years, in addition to surgical procedures like tooth scaling and root planning, various methods such as medication delivery into the bone defects and grafting have been employed to address these issues. Grafting, which involves introducing donor bone tissue into the bone defects, aims to fill the cavities around teeth with a specific material or tissue, enabling new bone formation. Essentially, the grafting material serves as a scaffold, maintaining the space required for new bone growth while preventing soft tissues (gingivae) from invading the bone defect. An essential factor determining the efficacy of this process is the seamless connection between the grafting material and the bone defect, ensuring that no gaps are left behind. This underscores the necessity for custom-designed scaffolds that precisely match the anatomical intricacies of the bone cavity to be filled, preventing the potential formation of gaps that could promote the proliferation of soft tissues.

To create such a customized scaffold, it is imperative to possess a highly detailed 3D representation of the bone defect. This detailed representation ensures that the resulting scaffold design closely aligns with the ideal anatomical characteristics of the bone defect. In this context, this study tests the workflow already proposed in our previously published research [12,13] for CBCT-based 3D modeling of periodontal defects (i.e., alveolar bone and teeth around bone loss of a patient’s oral cavity) and 3D-designed patient-specific scaffolds (bone grafts) for three cases of patients diagnosed with periodontitis and goes one step further by 3D printing these models with two different 3D printers, an FDM printer and a bioprinter. The suggested workflow has effectively produced 3D models of alveolar bone and teeth around periodontal defects and designed 3D-printed patient-specific scaffolds using bioabsorbable materials for periodontal regeneration for all three patients, proving its applicability for designing personalized bone grafts. The steps of the workflow are easy to follow using medical-image-editing software and 3D-editing tools. The suggested workflow may result in creating an exceptionally precise 3D digital representation of a periodontal scaffold within a span of fewer than two hours, utilizing CBCT data. This feature renders it a cost-efficient option for producing intricate digital 3D models of grafts. The 3D printing of the designed models with FDM also confirmed the accuracy and effectiveness of the proposed methodology, as the quality and precision of the developed scaffolds were significantly high. Specifically, in all patient cases, the scaffolds exhibited a precise fit to the periodontal defects, with slight discrepancies noted based on the specific printing profile employed. In detail, the results demonstrated that the standard printing profile emerged as the optimal choice in the FDM-based printing process, considering the required printing time, the material needed, and the application of scaffolds to the periodontal defects of each patient. On the other hand, bioprinting was not as effective as the FDM in terms of scaffold resolution and accuracy, which depend both on the printing material and the shape complexity of the required scaffold.

While our study utilized actual patient CBCT radiological data to create realistic anatomical models of osseous defects, we were unable to directly compare the accuracy of these models to the gold standard, namely the appearance of the osseous defects in the actual patients. This represents a limitation of our study. Currently, our research team is collaborating with clinical researchers to assess the accuracy of the resulting models and scaffolds in real patient scenarios. Looking ahead, as biotechnology progresses, we anticipate the resolution of ethical and regulatory issues associated with these advancements.

The scaffolds produced through the proposed CBCT-based 3D-design approach stand apart from most of 3D-printed grafts documented in the existing literature, like those outlined in references [29,30,31]. The key distinction lies in the fact that the latter scaffolds are typically pre-designed or possess a standardized structure, disregarding the intricate morphological structure of the actual damage. In contrast, all scaffolds generated and printed using our suggested approach are personalized bone grafts, precisely tailored to fit the specific hard tissue contours of the patients’ periodontal defects. Hence, our study incorporates advanced CBCT-based 3D-modeling techniques to precisely capture the unique morphology of periodontal defects, ensuring accuracy in scaffold design. Unlike pre-designed scaffolds, our personalized 3D-printed bone grafts account for individual variations in defect size, shape, and location, optimizing the potential for successful periodontal regeneration. The integration of patient-specific data obtained from CBCT scans enhances the fidelity of our scaffold designs, facilitating a tailored approach to periodontal therapy. By prioritizing anatomical precision and patient-centric care, our methodology addresses a critical gap in the existing literature by offering customized solutions for periodontal-tissue engineering. These advancements underscore the transformative impact of CBCT-guided 3D design on the development of next-generation regenerative therapies for periodontal diseases. Furthermore, while a handful of prior research articles have addressed the concept of patient-specific scaffolds using CBCT data, as seen in [43,44,45], they do not divulge technical specifics about the scaffold design process, a focal point of our study. This gap in the literature highlights the need for a more detailed understanding of the scaffold design process to ensure reproducibility and reliability across studies. By meticulously elucidating the technical intricacies of scaffold design using CBCT data and the 3D-printing parameters of the scaffolds, our study not only advances the current knowledge base but also provides a robust framework for future investigations in the field. Additionally, our focus on technical specifics contributes to establishing a standard methodology for designing patient-specific scaffolds, thereby facilitating comparisons between different studies and fostering a more cohesive research landscape. Furthermore, by transparently documenting the scaffold design process, we empower fellow researchers to build upon our methodology and explore innovative avenues in periodontal regenerative medicine. This comprehensive approach not only enhances the scientific rigor of our study but also catalyzes further advancements in the field of personalized scaffold design and 3D printing for periodontal therapy. What is more, the novelty of our design hinges on the capability of our proposed workflow to represent osseous defects with a remarkable level of detail, allowing for the design of scaffolds that closely align with these defects with high precision. Other similar processing algorithms may offer sufficient detail for gross anatomical models rather than fine structures like periodontal bone defects. This level of detail and precision in representing osseous defects sets a new standard in periodontal regenerative medicine, promising enhanced treatment outcomes and patient-specific interventions that cater to the intricacies of individual cases. Therefore, this research is expected to stimulate further exploration and clinical applications in the domain of periodontal regenerative medicine.

## Figures and Tables

**Table 1:** Printing settings of the utilized printing profiles for the FDM technique.

	Print Profile		
	Standard	Fast	Fine
Base print speed (mm/s)	60	70	50
Travel speed (mm/s)	80	100	70
Fill density %	15	10	20
Top solid layer	4	6	3
Bottom solid layer	3	6	3

**Table 2:** Printing settings of all scaffold variants for both materials used for the 3D-bioprinting process.

	PLA Material			PCL Material		
	Printing Temperature (°C)	Printing Speed (mm/s)	Printing Pressure (KPa)	Printing Temperature (°C)	Printing Speed (mm/s)	Printing Pressure (KPa)
Scaffold 1— Patient 1	177	5	120	58	6	600
Scaffold 2— Patient 1	177	4	100	176	5	100
Scaffold 1— Patient 2	180	17	180	180	17	180
Scaffold 2— Patient 2	176	12	120	176	12	120
Scaffold 1— Patient 3	176	10	100	61	8	660
Scaffold 2— Patient 3	176	3–8	120	61	8	690

**Table 3:** Number of triangles and size of the 3D model of hard tissues for Patient 1.

3D Model of Hard Tissues	Number of Triangles	Size (MB)
Before processing	1,029,051	50.2
After processing	417,566	20.4

**Table 4:** Print time and material required at FDM printing, for each examined profile for Patient 1′s scaffolds.

		Standard	Fine	Fast
Scaffold 1	Print time (min)	7.00	11.00	4.00
	Material (gr/m)	3.00	3.50	3.00
Scaffold 2	Print time (min)	5.00	9.00	3.00
	Material (gr/m)	2.88	3.11	2.88
Alveolar Bone	Print time (min)	43.00	78.00	27.00
	Material (gr/m)	2.98	3.28	2.97

**Table 5:** Number of triangles and size of the 3D model of hard tissues for Patient 2.

3D Model of Hard Tissues	Number of Triangles	Size (MB)
Before processing	337,080	16.5
After processing	243,910	11.9

**Table 6:** Print time and material required at FDM printing, for each examined profile for Patient 2′s scaffolds.

		Standard	Fine	Fast
Scaffold 1	Print time (min)	3.0	4.0	2.0
	Material (gr/m)	3.00	3.10	3.00
Scaffold 2	Print time (min)	2.0	3.0	1.0
	Material (gr/m)	2.34	2.34	2.33
Alveolar Bone	Print time (min)	21.0	38.0	13.0
	Material (gr/m)	2.44	2.99	2.97

**Table 7:** Number of triangles and size of the 3D model of hard tissues for Patient 3.

3D Model of Hard Tissues	Number of Triangles	Size (MB)
Before processing	458,421	22.4
After processing	402,922	19.7

**Table 8:** Printing time and material required at FDM printing, for each examined profile for Patient 3′s scaffolds.

		Standard	Fine	Fast
Scaffold 1	Printing time (min)	6.00	11.00	4.00
	Material (gr/m)	2.90	3.00	2.90
Scaffold 2	Printing time(min)	5.00	7.00	3.00
	Material (gr/m)	2.83	3.20	2.33
Alveolar Bone	Printing time (min)	41.00	75.00	25.00
	Material (gr/m)	2.98	3.90	2.97

## Data Availability

No new data were created or analyzed in this study. Data sharing is not applicable to this article.
